# Development, Testing, and Implementation of the Belgian Patient Reported Experience Measure for Pancreatic Cancer Care (PREPARE) Project: Protocol for a Multi-Method Research Project

**DOI:** 10.2196/29004

**Published:** 2022-06-06

**Authors:** Katrien Moens, Marc Peeters, Marc Van den Bulcke, Mark Leys, Melissa Horlait

**Affiliations:** 1 Cancer Centre Sciensano Brussels Belgium; 2 University Hospital Antwerp (UZA) Antwerp Belgium; 3 Organisation, Policy & Social Inequalities in Healthcare Research Group (OPIH) Vrije Universiteit Brussels Brussels Belgium

**Keywords:** patient-centered care, quality of health care, interdisciplinary research, decision-making, pancreatic cancer, quality, outcome, assessment, cancer, pancreas, development, testing, implementation, patient-reported, experience, protocol, participatory medicine

## Abstract

**Background:**

Patients with pancreatic cancer do not feel involved in the development of their treatment and care plans. In Belgium, these plans are decided on during multidisciplinary team meetings. However, limited time is spent on the discussion of the preferences of the patient during these meetings. This research project aims to develop a patient-reported experience measure (PREM) for pancreatic cancer and assess if its use can support collaborative treatment decision-making.

**Objective:**

This paper aims to outline the protocol for a multi-method research project to improve person-centered pancreatic cancer care in Belgium. Three subobjectives are pursued: (1) to develop a PREM to assess the experiences of care-related aspects in pancreatic cancer care, (2) to validate the PREM, and (3) to develop and evaluate an educational intervention to support the use of the PREM’s results.

**Methods:**

For the development of the PREM, an exploratory mixed methods study design will be used. The study will start with a survey followed by a telephone interview involving patients with pancreatic cancer and digestive oncology health care professionals. Study two is the testing of the content and construct validity of the PREM. Study three involves the implementation study according to the Medical Research Council framework of a complex intervention introducing the PREM in practice. The effectiveness of the intervention will be investigated using a pragmatic randomized controlled trial study design.

**Results:**

The protocol presents the entire structure of the research project. Ethics approval to conduct the exploratory mixed methods study (objective 1) has been obtained, and recruitment has started since January 2022.

**Conclusions:**

The poor prognosis of patients with pancreatic cancer should not be considered a hurdle to not study this patient population group. Involving patients in the research and decision-making processes early on is key. This project aims to realize a scientifically sound research process providing research outputs that can easily and timely be implemented in the care trajectory of patients with pancreatic cancer. This research project will also lead to recommendations on how to involve patients with pancreatic cancer and how the methodology of this research project can be translated to other patient groups.

**International Registered Report Identifier (IRRID):**

PRR1-10.2196/29004

## Introduction

### Background

Consensus grows that patients’ needs and experiences should be at the center of quality improvement initiatives. Person-centered care emerges internationally as a key indicator of the quality of (value-based) health care [1].

Person-centeredness is rooted in the definition of “patient-centeredness.” The standard definition of Stewart et al [2] states that patient-centeredness encompasses six interconnecting dimensions: exploring both the disease and the illness experience, understanding the whole person, finding common ground between the physician and patient, incorporating prevention and health promotion, enhancing the doctor-patient relationship, and “being realistic” about personal limitations and issues such as the availability of time and resources [2]. High levels of positive patient experiences are associated with higher levels of adherence to recommended prevention or treatment processes, better clinical outcomes, a better patient safety culture within hospitals, and less unnecessary use of health care [3].

Psychometric solid patient-reported experience measures (PREMs) are used as tools to assess person-centered care [4]. PREMs measure the patient’s experiences with health care services based on validated questionnaires. Greenalgh et al [5] frame the positive influence of well-implemented routinely collected patient-reported measures on patient health outcomes including their effects on doctor-patient communication, monitoring of the treatment response, detection of unrecognized problems, patients’ health behavior, and clinician’s management of the patient [5]. One other important benefit that PREMs bring along is their ability to enrich the practitioner-patient conversations in various ways; for example, the PREM’s results can be used to help support the patients’ reflection and expression during consultations [4].

### PREMs in Oncology

A recent systematic review concludes that oncology PREMs with good psychometric characteristics are lacking; most identified PREMs scored low on reliability, effectiveness, content, and construct validity [6]. General PREMs for all types of cancer are not recommended. The review argues that the specificity of measures and responsiveness of a PREM can be realized by developing a PREM for a specific type of cancer [6]. In 2019, a British pancreatic cancer survey was developed to measure the needs and experiences of patients with pancreatic cancer and to assess the services provided by the charity Pancreatic Cancer UK [7]. This survey is used for cross-sectional measurements [7].

Besides methodological issues, the use and impact of PREMs in clinical practice remains relevant. Furthermore, more knowledge grounded in research on how and when to introduce PREMs data during consultations is needed [4]. Some evidence identified critical moderating mechanisms to implement patient-reported data such as providing multiple moments of feedback over a period of time [8-11]; addressing different stakeholders (physicians, nurses, allied health care professionals, as well as patients) with simple, clear, graphical, and longitudinal meaningful interpretation of the measurement results; providing training for both health care professionals and patients; and offering decision-making aids, clear management plans, and clinical pathways including referrals [12-14]. Hampering factors are classified as people-based barriers, questionnaire-based barriers, and barriers related to access and interpretation of PREMs data.

### Rationale for a Pancreatic Cancer PREM

In 2018, a total of 2024 of the 70,468 (~3%) Belgian people diagnosed with cancer have pancreatic cancer [15]. It ranks as the 10th most common cancer type in Belgium [15]. Pancreatic cancer is often detected at a late stage frequently combined with metastases or growth in the surrounding tissue [16]. Only 12.4% of patients live for more than 5 years [15]. For the majority of patients, the tumor cannot be removed. Palliative treatment with chemotherapy, whether or not supplemented with radiation, is most often considered [17].

An assessment of the organization of pancreatic cancer treatment and care in Belgium mentioned the lack of concentration of expertise in hospitals to treat and care for patients with pancreatic cancer [17]. In July 2019, expertise centers were formally recognized for, for example, complex surgical interventions. At the same time, quality indicators to monitor care and treatment were identified: the time between confirmed diagnosis and the start of the treatment; 1-, 3-, and 5-year observed survival; and relative survival. It is essential to also assess patient experiences, but a pancreatic cancer PREM is not yet available in Belgium. A specific instrument measuring the experiences of the patient’s entire care trajectory (from diagnosis to follow-up) will support the quality improvement process.

Overall, patients with pancreatic cancer are underrepresented in psychosocial research owing to the limited prognosis of the disease [7]. Patients with pancreatic cancer do not feel involved in the development of their treatment and care plans mainly because they are stressed by the life-threatening nature of their condition [7,18]. Nonetheless, this is a population with potentially severe needs [18]. Hence, the specific PREM—the results of which are immediately used to improve care and treatment delivery—could also be highly relevant, additionally for strengthening a team-based approach [18].

Pancreatic cancer treatment and care requires a multidisciplinary collaboration including professionals directly involved with the care of patients with pancreatic cancer such as digestive oncologists, nutritionists, psychologists, (digestive) oncology nurses, social assistants, physiotherapists, and occupational therapists [17]. Timely and appropriate care can only be delivered when these health care professionals work as a multidisciplinary team [17].

A multidisciplinary team is defined as two or more health care professionals interacting in a dynamic and flexible way working toward collectively agreed goals [19]. An often used tool to coordinate activities and decision-making processes are team meetings [20]. In Belgium, multidisciplinary team meetings in oncology take place in different formats; that is, formally regulated and financed multidisciplinary oncology consultations (MOCs), patient ward rounds, and ward meetings [20]. The benefits of multidisciplinary team meetings are well documented; for example, their ability to realize better team performance after case discussion being one of them [21]. However, one can question whether these MOCs are truly multidisciplinary, considering all types of patient needs [21]. Notwithstanding, a recent study found that in the context of MOCs, these in fact do not show a true multidisciplinary character [20]. This has mainly to do with physicians taking the upper hand during MOCs, mostly discussing biomedical information leaving nurses and psychologists with limited time to exchange information related to patient preferences and psychosocial aspects. This negatively impacts the implementation of treatment recommendations; for example, making nonholistic treatment recommendations [22,23]. Horlait et al [20] argue that the findings of their study can be used as a basis to design and implement interventions aiming to reinforce the inclusion of psychosocial information and patient preferences during MOCs [20]. This research project takes this recommendation on board, aiming to study if, for example, an intervention entailing the inclusion of PREM results during MOCs or other consultation settings can improve the delivery of person-centered pancreatic cancer care.

### Study Purpose

Within this research project, three subobjectives are pursued:

1. To develop a PREM measuring the experiences of care-related aspects in pancreatic cancer care;
2. To validate the PREM; and
3. To develop and evaluate an (educational) intervention to support the use of the PREM results in clinical multidisciplinary practice.

## Methods

### Design

This study is based on a multi-method approach combining qualitative and quantitative research methodologies. Three sequential studies are planned where the results of a step feeds into the next substudy.

### Objective 1: Development of the PREM

An exploratory mixed methods study design will be used to accomplish objective 1. The development of the PREM items starts with a systematic exploration of opinions on items to take into account by (a) patients with pancreatic cancer and their relatives and (b) health care professionals working in digestive oncology. The study will start with a (quantitative) survey followed by a (qualitative) telephone interview study. This latter strategy proved to be effective in other studies [4].

In the survey, respondents will be asked to assess the relevance of a list of items to include in the PREM using a Likert-based scale from 1=Very Important to 5=Very Unimportant. These items are extracted from the literature and the evidence with regard to needs and experiences of patients with pancreatic cancer. This survey will be piloted first to make sure that all items and the information in the supporting documents are well understood. Participants of the survey will be asked if they want to take part in a follow-up telephone interview, seeking more qualitative in-depth information about their vision on the importance of measuring patient experiences, which factors could influence this process, how and in which consultation setting these PREM results should be used, and on which time points PREM completions should be scheduled. The interviews will be semistructured, leaving sufficient leeway and flexibility to adapt to participants’ contribution, but structured by means of an interview guide topic list. These topics are also extracted from the evidence regarding barriers and facilitators when implementing the PREM’s results. The topic list will also be piloted first. The number of interviews will be defined by principles of saturation; that is, the moment on which no new data will be generated [24].

Respondents will be recruited via all Belgian digestive oncologists who treat patients with pancreatic cancer and who are in charge of multidisciplinary health care provision. These oncologists will receive an email asking to participate in the study and to help with the recruitment forwarding this call for participation to other digestive oncology health care professionals and patients with pancreatic cancer.

The data from the surveys will be descriptively analyzed, and an item will be included in the PREM if at least 75% of the respondents gave it an average rating of 2 or less on the Likert scale of importance. This 75% consensus determination is based on the recommendation of Keeney et al [25]. The list of domains that will eventually be consented on will then be reformulated to items for the PREM. These are then further evaluated in the validation study. The aim is to have at least 100 participants, which is a number suggested in the study by Boulkedid et al [26] to make sure a solid consent is realized [26].

The qualitative data generated via follow-up telephone interviews will be thematically analyzed using a grounded theory approach in which the principles of inductive reasoning will be applied, resulting in labels supported by quotes of the interviews [27]. This methodology allows us to analyze in a descriptive, explorative, and detailed way focusing on different research questions at the same time. The same analysis will be performed by two researchers; that is, investigator triangulation will be realized to reduce researcher’s bias when looking at the same data and when defining the themes [28]. The results of this qualitative analysis will provide input for the implementation intervention that will be developed, piloted, and tested as part of objective 3. All these data will be anonymized, and possible respondent-identifying information will be deleted from the interview transcripts. The audio files will be stored on a password-protected server.

### Objective 2: Validation of the PREM

Validation of the PREM will take place in several steps. The *content validity* of the PREM will be tested through focus groups (maximum of 7 persons per focus group) or individual interviews with patients with pancreatic cancer. They will be asked to assess the items in terms of the formulation or chosen terminology, the response scale, and the relevance of each item. After this, the stability of the items will be examined by means of a test-retest of the PREM. This involves a limited number of patients with pancreatic cancer, who will be asked to complete the survey presenting the PREM on two different time points. The construct validity will be tested on the PREM data set that will be generated via the PrCT (see objective 3) and by means of an exploratory factor analysis (EFA) that will be executed on the trial data. This will allow us to investigate how many dimensions can be determined in a construct and it will help to reduce items because items that have no contribution to the factors can be deleted [29]. Principal components analysis will be the statistical measure used. To be able to perform an EFA, a minimum of 100 patients is necessary [30].

The COSMIN (COnsensus-based Standards for the selection of health status Measurement INstruments) checklist will be used to assess the methodological quality of the PREM throughout its entire development and validation process [31]. This checklist specifies the standards related to the design of a PREM development study and the nine measurement-related property standards; that is, content validity, structural validity, internal consistency, cross-cultural validity or measurement invariance, reliability, measurement error, criterion validity, hypotheses testing for construct validity, and responsiveness.

### Objective 3: Testing the Effectiveness of the PREM Implementation Intervention

The results of the primary inductive analysis of the follow-up telephone interviews will be integrated in the framework of the development of a complex intervention according to the Medical Research Council (MRC) framework realizing a framework analysis. The results of this framework analysis will help identify the key elements for an intervention to implement the PREM. This proposed intervention will be presented to patients with pancreatic cancer and their health care professionals via focus groups aiming to explore its feasibility and to provide consent on the prefinal content of the intervention. Before testing the effectiveness of the PREM implementation intervention using a PrCT study design, a pilot study will be carried out. We chose a PrCT design because we will study the effectiveness of the intervention in a real-world setting or direct practice, and the results will be used to inform treatment effectiveness and health care decisions [32]. The intervention group will be the consultation setting using PREM results with, for example, facilitating (educational) interventions. The control group involves the standard consultation setting. We will use effectiveness criteria as proposed by Chen et al [7] (the effects it has on the treatment response; the changes to patient health behavior; the changes to clinicians’ management of the patient; and the improvement of health outcomes) as well as the experience of the patients.

The PrCT will follow the steps as specified in the Consolidated Standards of Reporting Trials 2010 checklist. This checklist indicates the aspects such as trial objectives, trial design, interventions, outcomes, sample size, randomization, blinding, statistical methods, participant flow, recruitment, baseline data, and numbers analyzed, which will need to be reported when carrying out the trial.

The PREM’s implementation intervention will need to be flexible enough to be used and adapted to each specific practice; hence, we can consider it a complex intervention [33]. In this perspective, we will use the MRC Framework for the development, evaluation, and implementation of complex interventions as our general guidance throughout the research project [33].

**Figure 1:**
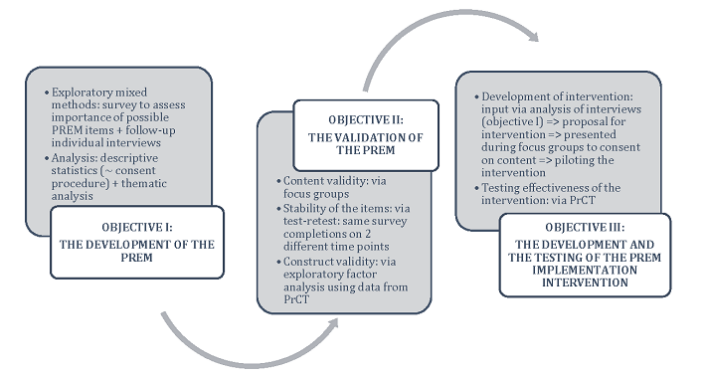
Diagrammatic representation of the steps and methods within the Patient Reported Experience Measure for Pancreatic Cancer Care (PREPARE) research project. PrCT: pragmatic randomized controlled trial; PREM: patient-reported experience measure.

Figure 1 below visualizes the different steps within the research project.

### Ethical Considerations

Ethics approval to conduct the exploratory mixed methods study (objective 1) has been obtained (B14320200000170) from the Medical Ethics Committee of the University Hospital Brussels that will be the central committee. If necessary additional ethical approval will be obtained from the participating hospitals.

## Results

The protocol presents the entire structure of the research project entailing three different studies each feeding into each other. For now, a steering group has been composed with representatives of the Belgian federal government of Public Health and Social Affairs, the National Institute for Sickness and Disability Insurance, the Belgian College of Oncology, Sciensano, two digestive oncology specialists, and the director of the Belgian pancreatic cancer community. This steering group gives direction to the research project and has already met and reviewed the research protocol, the survey and the interview guides that will be used for the study of objective 1. The tools (ie, survey and interview guides) that will be used within study 1 have been piloted in a group of 3 patients with pancreatic cancer and the health care professionals of the steering group. During this pilot, we asked these patients and health care professionals to complete the survey and read through the interview guides. After this, we explored their experiences in reference to the understanding of the items, the length of the survey and guides, and if additions were needed. Recruitment for objective 1 via the recruitment leaflets that were sent via email to the digestive oncologists has started since January 2022.

## Discussion

This paper presents a research protocol on the development and implementation of a PREM for patients with pancreatic cancer. Since person-centered care is one of the gold-standard practices, we wanted to understand how this can be strengthened. PREMs are measurement tools to assess the person-centeredness of care delivery. The project aims to study how a dedicated PREM for patients with pancreatic cancer can support collaborative treatment decision-making.

The choice of patients with pancreatic cancer is mainly grounded in the underrepresentation of patients with pancreatic cancer in psychosocial research [7]. Moreover, patients with pancreatic cancer do not feel involved in the development of their treatment and care plans [18]. The poor prognosis of patients with pancreatic cancer should not be considered a hurdle to study this patient population group. In particular, for this group, research outputs that can easily and timely be implemented in the care trajectory of patients with pancreatic cancer are needed.

This research project takes place in Belgium where decision-making processes on care and treatment plans take place in different types of multidisciplinary team meetings, but where treatment preferences of the patient are not very well taken up [20]. This observation calls for interventions that support and facilitate the patient’s voice during consultations. Moreover, in Belgium, a PREM for pancreatic cancer is not available. Developing a PREM incorporating the experiences of patients with pancreatic cancer adapted to the local context increases the specificity of the measurement tool and consequently its ability to capture the true situation of this patient group. The study is research-oriented but uses iterative involvement cycles of all stakeholders to develop the PREM item list and the intervention. One of the strengths of this research project is the involvement of patients with pancreatic cancer as well as oncology and digestive health care professionals, taking into account and comparing their perspectives. If discrepancies occur, it will provide us insight on aspects that should be considered when developing the implementation plan of the PREM; that is, it will help us assess the feasibility. By involving health care professionals and patients together with their family in the development, validation, and implementation process of the PREM, a common ground for the necessity of patient experience measurements will be achieved. The bottom-up approach to develop an (educational) intervention to introduce PREM results in the collaborative treatment decision-making process also makes the research project vigorous.

To prevent methodological errors, some particular quality control mechanisms and tools are used. These will strengthen the robustness of the research design. Throughout the entire research project, special attention will be paid to pilot the data collection instruments, such as surveys and interview guides, before effectively using them. User-friendliness and unambiguous understanding are aspects that, for example, will be evaluated during the piloting. Impact assessment tools with regard to patient engagement will be used to critically evaluate the way we involved patients and if the predicted aim of their involvement is achieved. The steering group will meet on critical moments throughout the research project to guard if the focus of the research project is retained.

The project will end up with recommendations on how to involve patients with pancreatic cancer in shared decision-making processes, on the international relevance of the findings and on how the methodology used can be translated to other patient groups.

## Abbreviations

COSMIN: COnsensus-based Standards for the selection of health status Measurement INstruments
EFA: exploratory factor analysis
MOC: Multidisciplinary Oncology Consultation
MRC: Medical Research Council
PrCT: pragmatic randomized controlled trial
PREM: patient-reported experience measure

## Data Availability

The data sets that will be generated during the different studies will be available from the corresponding author on reasonable request.
